# President’s Address

**Published:** 1891-10

**Authors:** M. H. Fletcher


### President's Address,

M. H. FLETCHER.

Before the Mississippi Valley Dental Society, Cincinnati, Ohio, March 10th, 1891.

*Mr. President and Gentlemen :—*

It is an agreeable duty which to-day falls to my lot to welcome you to the forty-eighth meeting of this association. It was suggested last year that the old ship be abandoned, but the mere mention of such a thing so aroused many of the members that a new interest, we hope, has been awakened, and we may expect that henceforth, not only the old time activity may be evinced, but also a new life, commensurate with the recent and rapid strides of our profession, may be instilled into the society. There seems to be no good reason why this should not be if the members do even a part of their duty, for our association enrolls a goodly number of talented workers and earnest investigators.

The history of the association has been given so often that it need not be repeated. But the growth and important advancement of our work, as a profession, must always be of interest, particularly since many of us remember the introduction and practical application of our greatest improvements.

The use of the rubber dam, cohesive foil and the dental engine, are well-nigh indispensable acquisitions to the practitioner of to-day. Porcelain teeth, crown and bridge work are the foundation of prosthetic dentistry, while the knowledge of bacteriology and germicides is to the dental pathologist the means by which many a diseased organ is restored to usefulness.

In reviewing our past progress we are emboldened to expect greater advancement in the future. The first and grandest of all these possible improvements is prophylaxis of dental caries. For if the prevention of decay of the teeth be possible (which we have faith to believe), who will deny such an attainment to be the acme of dental science. But while anticipating such a millenium, until it shall have become a happy realization, there is other work to be done.

A new substance for filling teeth is demanded, which must be as indestructible as gold, and as plastic as oxy-phosphate of zinc. Who of you will bring forth this material?

Who of you will devise a plan, or produce a substance which will universally alleviate pain during the cutting of sensitive dentine?

Again, there is yet to be invented a plastic base for the making of artificial dentures, more presentable than rubber, and more durable and tractable than celluloid, having the properties of strength and conductivity of temperature.

These are a few of the important points in which there is room for improvement and scope for the inventor. Such achievements, however, are slowly gained, and he who makes an advance in any line is taking us nearer the desired end. We have earnest workers who, by constant effort, are gradually bringing out new and valuable ideas. In fact, the successful practitioner is necessarily one who advances, since, to be successful he must be versatile, familiar with mechanics, physics and theory. Being able to turn readily from one to the other of these, his theory becomes practice and presents to us useful and advanced methods.

The work of Prof. W. D. Miller places before us in the most practical form yet devised the etiology of dental decay, and his theories are verified by evidence as strong as the microscope can reveal. Knowing a cause it naturally follows that we search for a remedy.

And now gentlemen, if we may depart from the conventional presidential address, we have a few suggestions to present with an apology that we could find nothing newer or less inviting for our subject than the treatment of pulpless teeth.

The late experiments of Prof. Miller have put us in the way of treating such teeth more intelligently, for the reason that we now have some facts as to what kind of germs we are dealing with. We do not question but that they have already been successfully treated by the intelligent practitioner, yet we have not been treating these conditions with a specific idea of the kind of life we are trying to destroy. I venture to suggest a plan (not entirely new) by which both patient and operator be saved time and annoyance. The theory is first, that gas-forming bacteria must be gotten rid of before these cases can be successfully disposed of, and that this condition can most quickly and conveniently be accomplished by the use of arsenious acid, or other strong germicides. I first venture the statement that there is no lesion of the tissues of the body (not self-limited) more amenable to treatment than the one in question. This is proved by the many and various kinds of treatment suggested and practiced. Many operators go so far as to say that they never lose a case. While they believe this to be true it is probable safer to say their loss is exceedingly small. Now, if it be so simple a process to treat these cases successfully it should also be a short process.

Dr. Geo. Cunningham, of Cambridge, England, gave three years ago, a paper and statistics on the treatment of such teeth with arsenious acid. He says of their curability: "The success of the operations seems mainly to depend on the old axiom, 'The cause being removed the effect ceases.' No observant operator can have failed to notice the inherent curability of abscessed teeth.

Who has not noticed the frequency of cicatricial tissue marking the existence of former fistulous tracts, even where the putrefactive contents had been allowed to remain? It is to this inherent property of spontaneous curability that we look for the relief and cure of all the injurious conditions arising from a putrescently diseased pulp."

Cunningham designates the arsenical treatment as the "Immediate method," and the other and longer procesess he calls the "Dressing method."

We do not claim originality in what we wish to present, except possibly the formula used, and the manner of its use. Dr. Cunningham gives the following:

Arsenious acid..........................................
Alcohol..........................................
Oil of clover..........................................

He reports a loss of only 3 teeth out of 512 treated.

Our own formular is,

Arsenious acid..........................................
Precipitated chalk..........................................
Glycerine sufficient to make a thick paste.

(Arsenic is only slightly soluble in water, 1 part dissolving in about 50 of water, and slightly more soluble in glycerine, so that only small portions of arsenic would be carried to the pulp chamber. No chemical union results from the mixture of arsenic with the chalk).

The latter formula I consider better because it is more easily manipulated, and in consequence is less dangerous to surrounding tissue: 1. Because the quantity is small by its admixture with chalk. 2. Because the quantity is easily measured by the eye, and being in a somewhat solid form is not likely to be forced through the apical foramen as a fluid might, while yet there is present an ample quantity to be a most thorough germicide. The dose of arsenic is from $\frac{1}{20}$ to $\frac{1}{4}$ of a grain: the stomach of an adult readily takes up this quantity. But in the use as we recommend there is probably not applied in any case more than $\frac{1}{40}$ of a grain, and this not in contact with soft tissue at any place unless it be forced through the foramen, and should this occur and there be a pus sack the $\frac{1}{30}$ or $\frac{1}{40}$ of a grain of arsenic would most probably be good treatment for it, acting upon the pus and destroying bacteria, and having done its work would become absorbed by the blood vessels with no destructive effect upon the tissues.

It may be used as follows: Take of the arsenical compound about $\frac{1}{2}$ grain, a portion about equal in size to $\frac{1}{2}$ grain of wheat, place this upon a glass slab and mix with it a drop of water from the end of the finger; then with a nerve broach covered with cotton mix the two thoroughly. This is introduced into the pulp canal, which has been previously cleansed, dried and washed with alcohol. Then introduce the arsenical fluid, being careful to get the canal thoroughly filled. Then dry again, and in the majority of cases, fill immediately. Teeth with small and tortuous root canals, into which it is difficult to explore or force a fluid, are probably better left with the dressing in, for a few days.

My statistics, 146 cases in the past eighteen months treated after this manner, show only two cases from which the dressing had to be removed to give relief from pain.

Another advantage in the use of the arsenic in this form is that small particles are carried with the solution into the root canal and remain there. The continual presence of such a germicide in the pulp chamber and canal is perfectly legitimate since the canal is in no way connected with the circulation after the death of the pulp and has no connection with the cementum which will allow the passage of any destructive agent.

These canals, if not filled nor sterilized, would most likely cause trouble sooner or later. According to Dr. Cunningham they may be safely left without filling if kept thoroughly antiseptic. The plan adopted was to cleanse the pulp chamber and larger root canals, then covering one side of a piece of paper with his arsenical compound he put it into the pulp chamber with the arsenical side toward the canals, then proceeded to fill upon this with cement, finishing with any other material desired.

The plan of using cotton saturated with carbolic acid and other antiseptics and filling over them is one of the oldest of the many methods, but have we not all been obliged to remove many such dressings after they have had time to become useless as antiseptics? The list of agents and plans for dressing root canals is too long to give now. According to our experience nothing so completely fills the requirements in every particular as arsenious acid. Fowler's solution might be used, but its strength is not always certain, and there is no crystal of the salt left in the canal to keep it antiseptic. We have also tried a solution of bichloride of mercury, and even with a 1 to 1000 solution trouble ensued. The powdered bichloride might be used in the same manner as the arsenic, but it is so much more soluble in water, and so dangerous to tissues that it is less desirable.

According to Dr. Miller some very active agent must be used to sterilize a pulp chamber. On page 97 of his late work on "Micro-organism" he says, "Attention has already been called to the fact that the dental pulp presents in a high degree the conditions essential to the formation of spores, and since spores possess high powers of resistance the antiseptic treatment of root canals is thereby rendered more difficult."

The necessity of destroying these germs is clearly set forth in the same work, page 111, where he says, "I would call especial attention to five different gas-forming bacteria, which invariably form large gas-bubbles in the gelatine, or tear it to pieces, as represented in the figure.

One of these bacteria, which generates considerable quantities of gas in albuminous substances, I found in the human fæces as well as in a gangrenous tooth-pulp. Its appearance in the latter place may help to explain the frequent occurrence of dental abscesses. If a tooth be filled before removing the necrotic pulp and sterilizing the root-canals, the gas formed will force itself through the foramen in the apex of the root, or carry particles of the putrid pulp along with it, causing irritation, if not immediate inflammation of the pericementum."

This it would seem, not only from recent investigation, but from the past experience of most practitioners, that the agents used have been such as would destroy organisms and spores, whether they were used with this idea or not. But in many cases the teeth were kept sore for weeks, and sometimes months, by the frequent introduction of steel points or worse remedies, each time introducing a few more bacteria, and explaining to the suffering patient that the case was a most difficult one to cure. On the other hand the rational treatment would seem to be that of removing the exciting cause by thoroughly sterilizing the pulp chamber and canal in the shortest possible time. These exciting causes may be foreign bodies, either fluids, solids or gases, all of which are accompanied with bacteria. Once these influences are removed the spontaneity of tissue is such that it will soon return to its normal condition. If the idea of the natural tendency of tissue to return to its normal condition can be thoroughly instilled into our minds, and also that we are simply to allow the living tissue to do what it is continually trying to do, then the treatment of abnormal conditions is greatly simplified. Time will be saved to patient and operator, and we will soon find ourselves in touch with natnre, which means immediate success in many cases where we now have much trouble.

Let us then keep in mind the old axiom, "The cause removed the effect ceases," and our trouble in the treatment of pulpless teeth is reduced to a minimum, and our patients saved both time and annoyance.

